# Population-based monitoring of cancer patient survival in situations with imperfect completeness of cancer registration

**DOI:** 10.1038/sj.bjc.6602323

**Published:** 2005-01-18

**Authors:** H Brenner, T Hakulinen

**Affiliations:** 1 Department of Epidemiology, German Centre for Research on Ageing, Bergheimer Strasse 20, D-69115 Heidelberg, Germany; 2 Finnish Cancer Registry, Institute for Statistical and Epidemiological Cancer Research, Liisankatu 21 B, FIN-00170 Helsinki, Finland

**Keywords:** cancer registries, prognosis, statistical methods, survival analysis

## Abstract

Selective underascertainment of cases may bias estimates of cancer patient survival. We show that the magnitude of potential bias strongly depends on the time periods affected by underascertainment and on the type of survival analysis (cohort analysis *vs* period analysis). We outline strategies on how to minimise or overcome potential biases.

Population-based monitoring of cancer patient survival is an important task of cancer registries (e.g. Berrino *et al*, 1995, 1999, 2003; Dickman *et al*, 1999; Talbäck *et al*, 2003). As with other cancer statistics, the validity of population-based cancer survival estimates depends on the quality of the cancer registry data. Most obviously, a minimum requirement is reliable follow-up of patients with respect to vital status. The validity of survival estimates may also depend on completeness of cancer registration (Monnet *et al*, 1998; Prior *et al*, 1998). In particular, selective underascertainment of patients with a good prognosis may lead to underestimation of cancer patient survival, whereas an opposite effect could result from selective underascertainment of patients with poor prognosis. The aim of this paper is to assess the impact of various patterns of incompleteness of cancer registration on population-based estimates of cancer patient survival in a quantitative manner.

## MATERIAL AND METHODS

### Database

Our analysis is based on data from the nationwide Finnish Cancer Registry whose true completeness (in terms of ascertainment of both incident cases and follow-up status) is known to be very close to 100% (Teppo *et al*, 1994). We included patients, aged 15 years or older, with a first diagnosis of one of the six most common forms of cancer in Finland between 1990 and 1999.

### Statistical analysis

**Figure 1:**
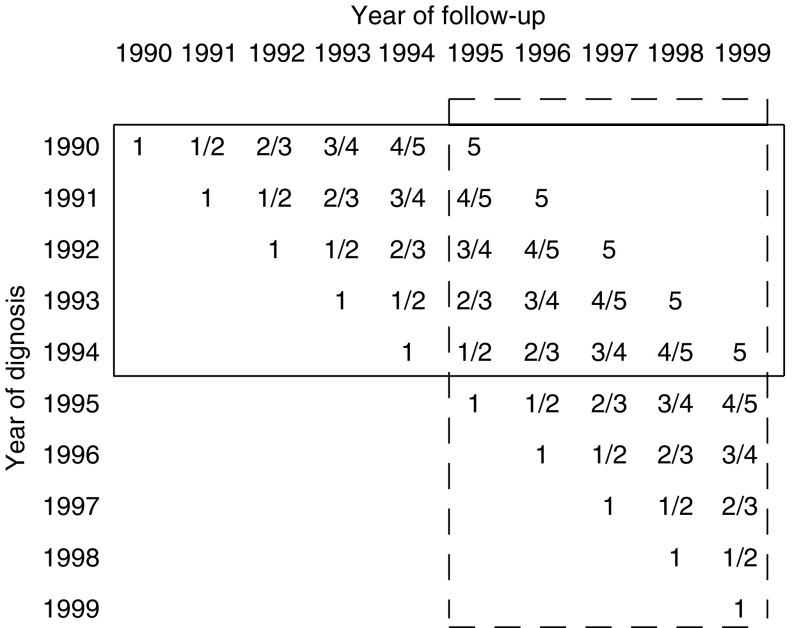
Database for calculating 5-year survival by the cohort method (solid frame) and the period method (dashed frame). The numbers within the cells indicate the years since diagnosis.

The impact of underascertainment of incident cases was assessed for 5-year relative survival rates (Ederer *et al*, 1961), which were derived using Hakulinen's (1982) method by two different approaches illustrated in Figure 1. With the first approach, 5-year survival rates were calculated for the cohort of patients diagnosed in 1990–1994 and followed with respect to vital status until the end of 1999 (solid frame). The second approach is the so-called period analysis, which has first been proposed a few years ago to provide more up-to-date estimates of cancer patient survival (Brenner and Gefeller 1996, 1997). Here, 5-year relative survival estimates for the 1995–1999 period are reported, which exclusively reflect the survival experience of patients during those years (dashed frame).

To assess the impact of incompleteness of registration either in the earlier or in the more recent years of the database, we carried out both a cohort analysis for the 1990–1994 cohort and a period analysis for the 1995–1999 period, assuming underascertainment of the following cases either in 1990–1994 or in 1995–1999 in different scenarios: (a) all cases, (b) only cases dying within 5 years following diagnosis and (c) only cases still alive 5 years following diagnosis.

Expected survival estimates for 80, 90, or 95% completeness of ascertainment of the specified patient groups were derived by weighted survival analyses, where a weight of 0.8, 0.9, or 0.95, respectively, was assigned to patients in these groups, and a weight of 1 was assigned to all other patients using a recently described SAS macro (Brenner *et al*, 2004a).

## RESULTS

Table 1 shows the numbers of patients by cancer site included in the analysis, as well as the estimates of 5-year relative survival obtained by the cohort method and by the period method from the full (presumably virtually complete) database. The most common form of cancer in Finland in 1990–1999 was breast cancer, followed by prostate cancer and lung cancer. Estimates of 5-year relative survival obtained by cohort analysis ranged from 81.6% for patients with breast cancer to 9.2% for patients with lung cancer. Period estimates were somewhat higher, with differences ranging from 7.4% units (prostate cancer) to 0.3% units (lung cancer). These differences reflect improvements in survival in the 1990s.

Unselective underascertainment of cases diagnosed in 1990–1994 would not affect cohort estimates of 5-year relative survival for patients diagnosed in those years. The 1995–1999 period estimates would be altered to some very minor extent (<0.3% units in all scenarios) by giving less weight to patients diagnosed in 1990–1994 compared to those diagnosed in 1995–1999. To save space, these results are not shown in a table.

As expected from theory, selective underascertainment of cases diagnosed in 1990–1994, who died within 5 years, would lead to overestimation of 5-year relative survival for the 1990–1994 cohort (see Table 2 ). For the most extreme scenarios, with selective underascertainment of 20% of these patients, 5-year relative survival would be overestimated by between 2.0% units (lung cancer) and 7.6% units (prostate cancer). The period estimates of 5-year relative survival for the 1995–1999 period would be much less affected by selective underascertainment of dying patients diagnosed in those earlier years.

By contrast, selective underascertainment of patients diagnosed in 1990–1994, who were still alive 5 years after diagnosis, would lead to underestimation of 5-year relative survival for the 1990–1994 cohort. Again, the bias would be quite small for lung cancer with its poor prognosis, and somewhat more pronounced for cancers with intermediate or more favourable prognosis. The period estimates of 5-year relative survival for the 1995–1999 period would again be much less affected by selective underascertainment of surviving patients diagnosed in those earlier years.

Obviously, underascertainment of cases diagnosed in 1995–1999 would not affect the survival estimates for the 1990–1994 cohort at all. The period estimates would also remain essentially unaffected if the underascertainment was unselective, that is, the same for patients who died and who did not die in 1995–1999. The period estimates for the 1995–1999 period could, however, be biased to some extent by selective underascertainment of patients diagnosed in that period (see Table 3 ). The potential bias would again be smallest for lung cancer with its poor prognosis, and somewhat more pronounced for cancers with intermediate or more favourable prognosis.

## DISCUSSION

Both cohort analysis and the more recently introduced period analysis are now well-established prototypes of population-based monitoring of cancer patient survival. Cohort analysis provides survival information on real cohorts of patients diagnosed within certain calendar years. Period analysis provides more up-to-date survival estimates, but these estimates pertain to patients diagnosed in a wider range of years of diagnosis and can less readily be linked to the patterns of early diagnosis and medical care during a defined time span (Brenner *et al*, 2004b). Hence, the choice between both methods would typically depend on the primary goal of the analysis.

This paper illustrates in a quantitative manner that the completeness of cancer registry data during various years may be an additional criterion for the choice of either method. For example, during the build-up phase of a new cancer registry, when completeness tends to increase over time, one might prefer calculation of period estimates over calculation of cohort estimates as the former may be less prone to bias by selective under-registration during the early years of registration. On the other hand, period estimates may be more prone to bias than cohort estimates if the completeness of the most recent available data is questionable due to delayed recording of some proportion of cases. In practice, however, the latter concern is typically relevant for a maximum of one or two of the most recent years for which data would be available, and one might still use a period analysis after excluding those years, either entirely or partly by means of a ‘hybrid’ type of analysis (Brenner and Rachet, 2004c). As period analysis has been shown to advance detection of trends in 5-, 10-, 15-, and 20-year survival rates by almost 5, 10, 15, and 20 years, respectively (Brenner and Hakulinen, 2002a), the slight loss of up-to-dateness that would follow from such a decision would still be almost negligible compared to the gain in up-to-dateness by the use of period analysis rather than cohort analysis. Furthermore, the magnitude of potential bias would have to be weighed against the often more substantial underestimation of current survival by cohort estimates (Brenner and Hakulinen 2002a, 2002b; Brenner *et al*, 2002c; Talbäck *et al*, 2004).

Which, if any, of the potential sources of bias may be relevant in a given study strongly depends on the specific circumstances under which a cancer registry is operating. Therefore, when choosing an analytic strategy, the specific circumstances of registration of the registries involved should be taken into account along with other aspects, such as up-to-dateness of cancer survival data.

When looking at our data, the following limitations should be considered. Results were presented for 5-year relative survival rates only, as these are the survival rates most commonly reported by population-based cancer registries. We also carried out analogous analyses for 5-year absolute survival rates. However, patterns were generally very similar, and they were therefore not shown separately to save space. Finally, we focused on very specific, relatively extreme patterns of entirely selective underascertainment of cases to illustrate the general principles. In practice, the impact of underascertainment of patients with relatively poor prognosis and of patients with relatively good prognosis would usually partly (and sometimes fully) cancel out, leading to smaller biases than those shown in our analysis.

Despite these limitations, our analysis illustrates the potential impact of incompleteness of cancer registration on various types of population-based monitoring of survival. The identified patterns could be valuable for decisions regarding the best analytic strategy in specific situations. The analyses also underline once more the crucial requirement of high levels of completeness for the use of population-based cancer registration.

## Figures and Tables

**Table 1:** Numbers and 5-year relative survival of patients diagnosed with common forms of cancer above 14 years of age in Finland in 1990–1999

		**5-year relative survival (%)**		
**Site**	***n* a**	**1990–1994 cohort**	**1995–1999 period**	**Difference (% units)**
Stomach	9262	25.9	28.8	+2.9
Colon	11 487	53.3	57.8	+4.5
Rectum	7715	50.9	54.9	+4.0
Lung	20 201	9.2	9.5	+0.3
Breast	29 859	81.6	83.2	+1.6
Prostate	22 491	66.5	73.9	+7.4

a Total number of patients in 1990–1999.

**Table 2:** Estimates of 5-year relative survival (%) for the 1990–1994 cohort and for the 1995–1999 period expected with various patterns and various levels of selective underascertainment of cases diagnosed in 1990–1994

		**1990–1994 cohort**				**1995–1999 period**			
		**Case ascertainment**				**Case ascertainment**			
**Selective underascertainment of**	**Site**	**80%**	**90%**	**95%**	**100%**	**80%**	**90%**	**95%**	**100%**
cases dying within 5 years	Stomach	30.7	28.1	27.0	25.9	30.6	29.7	29.2	28.8
	Colon	59.9	56.4	54.8	53.3	60.3	59.0	58.4	57.8
	Rectum	57.5	54.0	52.4	50.9	57.6	56.2	55.6	54.9
	Lung	11.2	10.1	9.6	9.2	10.5	10.0	9.7	9.5
	Breast	85.8	83.7	82.6	81.6	85.2	84.2	83.7	83.2
	Prostate	74.1	70.1	68.3	66.5	77.7	75.8	74.9	73.9
									
cases still alive after 5 years	Stomach	21.8	23.9	24.9	25.9	27.0	27.9	28.4	28.8
	Colon	46.9	50.2	51.8	53.3	55.2	56.6	57.2	57.8
	Rectum	44.6	47.9	49.4	50.9	51.9	53.5	54.2	54.9
	Lung	7.5	8.3	8.8	9.2	8.6	9.1	9.3	9.5
	Breast	77.0	79.5	80.6	81.6	81.0	82.2	82.7	83.2
	Prostate	59.0	63.0	64.8	66.5	70.2	72.2	73.1	73.9

**Table 3:** Estimates of 5-year relative survival (%) for the 1995–1999 period expected with various patterns and various levels of selective underascertainment of cases diagnosed in 1995–1999

	**1995–1999 period**				**1995–1999 period**			
	**Ascertainment of cases diagnosed and dying in 1995–1999**				**Ascertainment of cases diagnosed and surviving in 1995–1999**			
**Site**	**80%**	**90%**	**95%**	**100%**	**80%**	**90%**	**95%**	**100%**
Stomach	32.9	30.7	29.7	28.8	25.0	26.9	27.9	28.8
Colon	62.7	60.1	59.0	57.8	53.0	55.5	56.7	57.8
Rectum	59.4	57.1	56.0	54.9	50.6	52.8	53.9	54.9
Lung	11.2	10.3	9.9	9.5	8.0	8.7	9.1	9.5
Breast	85.3	84.2	83.7	83.2	80.8	82.1	82.7	83.2
Prostate	78.0	75.9	74.9	73.9	69.5	71.8	72.9	73.9
